# Acceptability of Hypertension Quality Indicators in Primary Care in South Africa: Exploratory Implementation Research

**DOI:** 10.3390/healthcare14121666

**Published:** 2026-06-11

**Authors:** Enos M. Rampamba, Stephen M. Campbell, Brian Godman, Johanna C. Meyer

**Affiliations:** 1 Department of Public Health Pharmacy and Management, School of Pharmacy, Sefako Makgatho Health Sciences University, Ga-Rankuwa 0208, South Africa; 210253700@swave.smu.ac.za (E.M.R.); stephen.campbell@manchester.ac.uk (S.M.C.); hannelie.meyer@smu.ac.za (J.C.M.); 2 School of Health Sciences, University of Manchester, Manchester M13 9PL, UK; 3 Antibiotic Policy Group, City St. George’s, University of London, London SW17 0RE, UK; 4 Strathclyde Institute of Pharmacy and Biomedical Sciences, University of Strathclyde, Glasgow G4 0RE, UK; 5 South African Vaccination and Immunisation Centre, Sefako Makgatho Health Sciences University, Ga-Rankuwa 0208, South Africa

**Keywords:** hypertension, quality of care, quality indicators, acceptability, healthcare professionals, primary healthcare, South Africa

## Abstract

**Background/Objectives**: Hypertension contributes to almost 5 million deaths annually, exacerbated by poor quality of care. Currently in South Africa, the quality of care for patients with hypertension is unknown, and there have been no agreed-upon quality indicators to measure this at the primary healthcare (PHC) level. Quality indicators have previously been developed to address this although their acceptability in PHC facilities in South Africa has not been established. Consequently, we aimed to explore the acceptability of these indicators, and their subsequent use for monitoring adherence to hypertension guidelines, as well as assess their availability and accessibility. Subsequently, identify recommendations for service-delivery changes to improve the uptake of evidence-based indicators into routine practice and the quality of hypertension care in South Africa. **Methods**: Questionnaire survey during semi-structured interviews with one key informant from 12 PHC clinics where the indicators had previously been tested. **Results**: Most facilities accepted hypertension quality indicators to improve hypertension management and patient outcomes. Ten clinics had assessed the facility’s quality of care, and all had access to hypertension guidelines. However, only two clinics had a hypertension-specific patient register. Concerns also included a manual data management system and nurse shortages. **Conclusions**: Large-scale application of quality indicators would facilitate evidence-based decisions for future use at PHC clinics to improve monitoring of adherence to hypertension guidelines and patient outcomes. However, this needs investing in electronic information management to improve data accuracy and management alongside a nursing workforce strategy. Replicating this study in other LMICs can provide comparable preliminary data within the region.

## 1. Introduction

More than 1.3 billion adults globally have confirmed high blood pressure [1], with some estimates suggesting that 1.28 billion adults in low- and middle-income countries (LMICs) have hypertension [2,3]. Hypertension prevalence rates are as high as 77.6% among patients with diabetes in Central Africa [4], with prevalence rates continuing to increase, exacerbated by increasing urbanisation coupled with sedentary lifestyles [5,6,7]. This includes South Africa, alongside high rates of underdiagnosed hypertension [8,9,10].

Despite considerable evidence concerning hypertension management and its role as a major cardiovascular (CVD) risk factor, control of blood pressure remains a challenge globally, especially among LMICs [1,5,6]. Overall, hypertension contributes annually to almost 5 million deaths globally. There is also increased morbidity associated with hypertension, which is exacerbated by the fact that only 10% or less of patients in Sub-Saharan Africa with hypertension currently meet their target blood pressure goals [5,7,11,12,13].

Key challenges across sub-Saharan Africa, including South Africa, include the current poor quality of care among patients with hypertension. This is mainly due to a lack of agreed, and consistently used, hypertension practice guidelines. Poor quality care is also worsened by a lack of agreed quality indicators to measure the actual care that patients are receiving to inform future proactive interventions [14,15,16].

Despite hypertension management being a priority in South Africa, and the government implementing multiple strategies including the development and dissemination of the South African National Strategic Plan (NSP) for the Prevention and Control of NCDs 2022–2027 (NSP for NCDs) as well as hypertension management guidelines, instigation of the Central Chronic Medication Dispensing and Distribution (CCMDD) system, the Ideal Clinic Project, and free services at the PHC level with the implementation of the national health insurance (NHI), the quality of care for patients with hypertension remains a challenge in the country [17,18,19,20,21,22,23]. Preliminary data from our previous study indicated gaps in the current quality of care for patients with hypertension in South Africa. Alongside this, there are currently insufficient data to apply potential hypertension quality indicators at PHC clinics to further highlight non-adherence to guidelines [23]. This is even more concerning for South Africa, where the rate of HIV/AIDS is high, and considering the recent increase in prevalence and death from hypertension among people living with HIV and AIDS in Sub-Saharan Africa [24,25,26,27].

These combined strategies are important for improving the quality of care for patients with chronic conditions at the PHC level in South Africa. However, accurate assessment of the quality of care, including its effectiveness in achieving planned patient outcomes in patients with hypertension among PHC clinics in the South African context, is lacking. This is important considering evidence of non-adherence to hypertension guidelines in South Africa, the high cost of healthcare and the implementation of NHI offering universal healthcare [17,20,21,28,29,30,31,32,33,34,35]. This is critical because quality improvement begins with understanding the baseline for which quality indicators are necessary to improve care, including guiding audits on adherence to hypertension guidelines [32,36,37,38]. This is imperative given that approximately 80% of South Africa’s population is managed in PHC facilities, current concerns with the management of patients with hypertension in the country, and to ensure that a target goal of 80% control of blood pressure among patients on treatment at the PHC level is achieved [11,19,23]. Our previous study found that only 53.2% of patients in public PHCs had controlled blood pressure [23].

Quality indicators enhance medical audit and quality improvement by identifying and providing important information about the quality of care for individual patients when they are mapped onto, and used, in routine data collection [39,40]. Such data, aligned with evidence-based hypertension management guidelines, would assist PHCs by providing a quality assessment of the care they currently provide. This should prompt healthcare professionals (HCPs) in the PHCs to consider management options for individual patients. This could include modifying patients’ lifestyle behaviours and addressing current non-adherence to treatment. The implementation of hypertension quality indicators would also necessarily improve the documentation of care provided to patients, a current concern in South Africa, by assisting in assessing and enforcing adherence to agreed-upon clinical guidelines, which, in turn, would improve patient outcomes [12,17,22,38,40,41].

Quality improvement requires a multifaceted approach with core elements. This includes competent human resources, evidence-based guidelines, electronic data management, medicines, devices, information systems, and adequate financing [42]. Some of these critical elements were identified as concerns in the previous study among PHC facilities in South Africa [23]. Improving data quality through disease-specific registers, e-health systems, and electronic health records, alongside excellence in healthcare structures and workforce planning, underpins the foundational elements of a quality healthcare system [42]. Electronic health record (EHR) data are a more immediate, actionable, and efficient, source of detailed longitudinal clinical information about individual patients [43,44]. Disease-specific patient registers are also important for the management of NCDs, such as hypertension, and for facilitating recall systems that invite patients to review their appointments [45,46]. Investing in electronic patient registers and health records is crucial for the successful monitoring of health programmes.

In our previous study, 45 quality indicators were developed, using the RAND/UCLA Appropriateness Method to assess the management of hypertension at the PHC level in South Africa [23,32] (Table 1). These indicators, documented in Rampamba et al (2025) [23], were subsequently found to be appropriate and feasible for monitoring hypertension management at the PHC level in South Africa [23]. As a result, providing standardised, evidence-based tools to measure the actual quality of care among patients with hypertension seeking care at PHC facilities in South Africa. However, only 22 could currently be applied and measured for ≥75% of the sample among PHCs in South Africa (Supplementary Table S1) [23].

The acceptability though of these indicators among PHC clinics in South Africa, where they will be applied, has not yet been established [47,48,49]. This is one of the requirements for testing quality indicators, and it was important to complement our previous work undertaken in the target clinics, which tested the applicability, feasibility, and measurability of the developed quality indicators (Table 1) [23,50,51]. This is necessary to guard against unintended consequences of quality improvement programmes and to assess their acceptability in the contexts in which they will be applied [47,48,49].

Acceptability is a subjective factor based on how people who deliver or receive a healthcare intervention perceive it [52,53,54]. An evidence-based approach to assessing acceptability should evaluate all three constructs: the Intervention Measure (AIM), the intervention Appropriateness Measure (IAM), and the Feasibility of Intervention Measure (FIM) [55]. Appropriateness is the relevancy or compatibility of the intervention (hypertension quality indicators) to address a specific challenge (poor quality of care) in the processes (hypertension management) [55]. Feasibility, on the other hand, refers to the extent to which the intervention can be implemented under a specific environment (PHC clinics) [55]. This emphasises the importance of embedding stakeholders’ experiences in any quality improvement project to ensure the quality assessment is applied and rooted in the context of coalface provision, as well as in the intentions perceived by the implementors [52,53,54]. The acceptability of quality improvement interventions in healthcare is crucial for their successful implementation [53,55,56]. There is currently no data available on the acceptability of potential hypertension quality indicators (Table 1) at the PHC level in South Africa.

Consequently, this study aimed to address this crucial gap by first exploring the prospective acceptability of these quality indicators and their use in monitoring guideline adherence to enhance their use in practice. Secondly, it aimed to assess the availability of, and access to, hypertension management guidelines among PHC clinics in South Africa. Thirdly, its aim was to identify recommendations for service delivery changes that may be needed in PHC clinics in South Africa to improve future uptake of the evidence-based indicators into routine practice and the subsequent quality of hypertension care. Assessing the acceptability of the quality indicators would provide information on their appropriateness and relevance within the available resources at the PHC level in South Africa. These findings can subsequently be used to help improve the care of these patients in South Africa and beyond, building on our previous findings in South Africa [23].

## 2. Materials and Methods

### 2.1. Study Design and Setting

This study employed an implementation research design focusing on evaluating the acceptability of hypertension quality indicators across 12 PHC clinics in a rural province in South Africa, where a previous cross-sectional study had evaluated the clinimetric properties of these quality indicators and assessed the quality of care at these clinics [23,39,48,57]. Whilst the questionnaire was based on the Theoretical Framework of Acceptability (TFA), the results were interpreted in line with the three acceptability constructs (Section 2.5, data analysis). Consequently, this study was a continuation of our previous work, intended to complement previous findings, and to provide preliminary data for future research, quality improvement projects, and policy development [23,55]. This is seen as important for the successful implementation of any hypertension quality indicators.

### 2.2. Study Population and Sample

The study population consisted of registered professional nurses, clinical practitioner nurses, and nurse facility managers with a primary care specialisation. In South Africa, registered professional nurses focus on comprehensive patient assessment and the implementation of care under the supervision of a medical doctor. In contrast, clinical nurse practitioners and primary care nurse specialists have autonomy in diagnosis, prescribing, and the independent management of patients across all presenting conditions at the PHC level [58,59]. All these professional categories are trained to function as generalist practitioners at the entry level and as specialist practitioners, depending on the area of specialisation and the category of care in which they are working, with the latter two requiring a post-basic qualification [58,59].

A total of 12 participants were included in this study. Purposive criterion sampling was used to select one key informant with specific knowledge of indicator use from each of 12 PHC clinics based on the following criteria: (i) the facility participated in the testing of the previously developed hypertension quality indicators; and (ii) the key informant must be a registered professional nurse, clinical practitioner nurse, or a nurse facility manager with a PHC specialisation managing patients with hypertension and willing to participate in the study [60,61].

The objectives and inclusion criteria of this study limited the number of facilities that could be included and, therefore, the number of participants. However, this does not affect the sample size for this study’s objectives. In exploratory implementation research, especially when empirical data are collected, and objectives are clearly defined, a sample of 12 participants is considered adequate for evaluating the acceptability of indicators among knowledgeable stakeholders to uncover the major categories of identified themes using simple content analysis [60,61,62]. Saturation in this study was interpreted as code saturation, intended to identify broader themes in acceptability, rather than meaning saturation, which may require a larger sample to understand and explain the different themes in the acceptability of different indicators [63]. Consequently, overall, the sample size was considered acceptable to meet the study’s objectives, mainly because it is homogeneous and addresses narrow objectives [63].

### 2.3. Data Collection Instrument

A semi-structured questionnaire was developed in line with previously recommended assessment tools and evaluations of the acceptability of retrospective or prospective quality improvement interventions [52,55,64]. The questionnaire had 14 questions. One question concerned the demographic characteristics of the participants; 7 (6 close-ended and 1 open-ended) questions examined the availability and accessibility of hypertension guidelines in the respective PHC clinics; and 1 open-ended question assessed the current quality of care for patients with hypertension.

A total of 4 questions adapted from previous studies were subsequently rated on a 5-point Likert scale to directly assess the prospective acceptability (AIM, IAM, FIM) of the devised quality indicators, with 1 open-ended question included to elicit any information, including input and concerns, that participants deemed necessary for implementing hypertension quality indicators at the PHC level in South Africa. A five-point Likert scale was chosen for its reliability in assessing acceptability [55,56,65]. These four questions were further categorised into three constructs that are directly linked to three implementation outcome measures, i.e., AIM, IAM, FIM, of interventions for which they are aligned to the TFA model [52,55]. The question of whether “the implementation of hypertension indicators would be fair to the facility” was used to assess the AIM. The question of “the implementation of hypertension indicators will improve the management of hypertension patients in your facility” was used to assess IAM. The last element of acceptability FIM was assessed by two questions of “implementation of the hypertension indicators will interfere with your other priorities and implementation of the hypertension indicators will be time-consuming for you”. The last two questions for assessing the FIM were used as intended, with no negative responses on the Likert scale. Only four questions were used to gain insight into participants’ acceptance of hypertension indicators, rather than to investigate the reasons for that acceptance in depth. This was important to provide evidence on the perceived effectiveness of the indicators and on the participants’ self-efficacy in implementing them.

The finalised questionnaire aimed to assess the acceptability of the indicators and their use in improving the quality of care within the South African PHC system [23,32,39,50,66]. Questions about awareness of and access to hypertension guidelines were included in this questionnaire to identify which indicators were necessary to assess challenges with guideline access and use, as this has been a concern across LMICs, including South Africa [54]. The questionnaire also included questions about quality assessments previously conducted at the facility to determine whether participants in the PHC clinics were aware of, and currently participated in, any quality improvement audits and interventions aimed at improving the future management of patients with hypertension. It was considered necessary to understand their experiences and knowledge of quality indicators and quality improvement interventions, which are important for assessing their acceptability.

Close-ended questions were self-administered by participants. They assessed opinions on the potential implementation of hypertension quality indicators in their clinics, including the burden of use, necessity, and potential effectiveness of the indicators in improving future care for patients with hypertension [56]. Responses to these questions were used to gauge participants’ general acceptance of the indicators and their implementation. A five-point Likert scale (ranging from “Strongly disagree” to “Strongly agree,” with a “Neutral” option) was again used to measure participants’ perceptions of the hypertension quality indicators [56,65]. Open-ended questions were administered by the interviewer, allowing for probing as necessary [50,65]. This was undertaken to gain insight into participants’ perceptions and experiences regarding potential changes, as well as the barriers and enablers to implementing hypertension quality indicators in practice. Open-ended questions were used to elicit any additional information that participants considered important that the questionnaire could not capture.

Pre-testing of the questionnaire was conducted at a clinic not included in the study population prior to data collection to assess its applicability to the current study setting [52]. The pre-testing was conducted face-to-face with five purposefully selected participants, including three clinical nurse practitioners, one registered professional nurse, and a manager with PHC specialisation. The questions were clear to the participants, and their responses demonstrated their understanding. The pilot study results also seemed to address the study questions fully. Consequently, no changes to the questionnaire were required before use in the main study.

### 2.4. Recruitment and Data Collection

Data were collected by the lead author (EMR) between January 2024 and April 2024. Participants were identified by code in the data collection tool (questionnaire) to prevent their identification during or after data collection. The facility managers at each of the 12 participating PHC facilities identified their nurses who manage patients with hypertension as the potential participants in the study. The researcher subsequently requested that either of them participate, typically the first nurse encountered, as they were introduced separately in their consulting rooms. In some clinics, facility managers, who also managed patients with hypertension, identified themselves as the person most suitable for participation in the study given its aims and objectives. This occurred when the manager was the only nurse managing patients with hypertension, or the most experienced nurse.

Potential participants were informed (written and verbal) of the aims and objectives of the proposed research project. They were provided the opportunity to ask questions and given adequate time to consider the research before possible participation. Potential participants were also not pressurised in any way to take part in the study and were informed that their names would not be revealed. Each participant subsequently signed a consent form before participation (Supplementary Table S2).

The semi-structured questionnaire was administered during the interview in a private room at the facility. The interviewer (EMR) asked the questions, provided clarity when required, and prompted when necessary, while participants scribed their responses on the questionnaire, allowing them to record their own views and recommendations. This approach enabled the verbatim transcription of open-ended questions.

### 2.5. Data Analysis

Responses to all questions were entered into a Microsoft Excel spreadsheet, including verbatim transcripts of participants’ responses to open-ended questions, and were coded by the researcher. A second researcher, who had considerable experience in both qualitative and quantitative research, reviewed and resolved discrepancies in data entry and coding by comparing the captured data with participants’ responses recorded in the data collection tools. Data from close-ended questions were summarised as frequencies.

Hypertension guidelines were considered available if a hard copy or electronic version was available, whereas guideline accessibility refers to the ready availability of the guideline to the practitioner during consultation.

Responses to Likert scale questions were subsequently categorised into three categories to improve analysis: “Disagree” (“strongly disagree” and “disagree” combined), “Agree” (“strongly agree” and “agree” combined), and “Neutral”. Responses to these three Likert scale categories were used to determine participants’ agreement or disagreement with the indicators and their implementation, with agreement interpreted as the acceptability of the indicators [52].

Responses to open-ended questions were transcribed verbatim, and the categories were generated in line with the identified themes. Descriptive categorisation of themes from content analysis was also undertaken. There were no pre-defined themes. The primary outcome variable (acceptability of the quality indicators and their implementation) was measured by aggregating the average percentage scores across the 4 questions that were rated on a 5-point Likert scale. This was undertaken to reflect participants’ perceptions, as measured by their collective average scores across all four questions that assessed the acceptability of the indicators in terms of their ethicality, coherence, and burden on participants. This was seen as necessary in order to determine the participants’ affective attitudes towards the hypertension indicators.

The acceptability of the hypertension indicators was determined by averaging the scores across the three acceptability constructs: AIM, IAM, and FIM. There is currently no internationally acceptable cut-off score for assessing acceptability. Hypertension quality indicators were considered acceptable if the average exceeded 50% [56,67].

The scores from the two questions about assessing the FIM were combined, while the scores of the other constructs remain as they are because only one question per construct was used. There were no specific reasons to use two questions for the FIM. Acceptability was interpreted as acceptance of the hypertension indicators, including their implementation at PHC clinics.

### 2.6. Ethical Approval

The study received ethical clearance from the Sefako Makgatho Health Sciences University Research Ethics Committee (Ethics reference no: SMUREC/P/93/2023:PG—9 March 2023). Permission to collect data from PHC facilities was granted by both the Limpopo Provincial Health Office and the Vhembe District Health Office.

## 3. Results

The results are presented in accordance with the study objectives, including participant demographic data.

### 3.1. Facilities and Participants

Participants included seven registered professional nurses and five clinical practitioner nurses, of whom three were facility managers with primary care specialisations and were responsible for managing patients with hypertension.

Table 2 presents the characteristics of the PHC clinics that were included in the study.

### 3.2. Acceptability of the Quality Indicators and Their Use in Monitoring Adherence to Guidelines

More than half of the participants agreed that implementing the hypertension quality indicators would improve the quality of hypertension management in their PHC facilities (Table 3).

**Figure 1:**
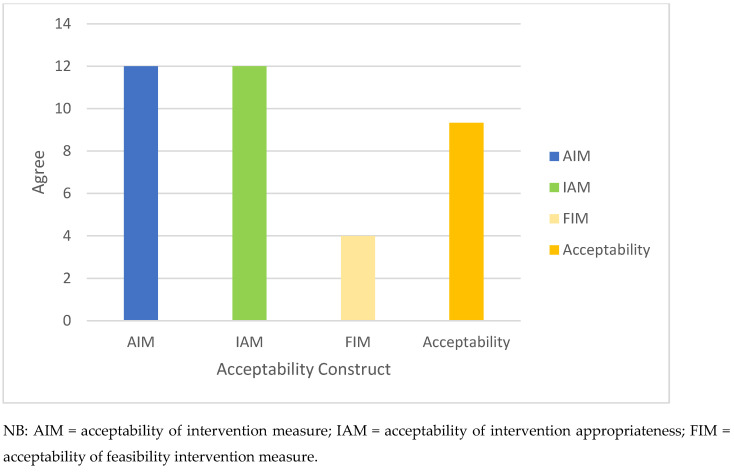
Acceptability of hypertension indicators.

Acceptability of the hypertension indicators was assessed as the average score across the three acceptability constructs (Figure 1).

One of the affective attitudes of the participants on the quality indicators and their implementation in relation to the current settings included:

“Hypertension quality indicators would complement the current processes.” (Participant 03).

Overall, the participants demonstrated approval of the hypertension indicators as an appropriate, feasible measure of the quality care of patients with hypertension in PHC clinic settings.

### 3.3. Availability of Hypertension Management Guidelines Across Participating PHC Clinics

Hypertension guidelines were considered available if a hard copy or electronic version was available at the PHC clinic. There were several guidelines for the management of hypertension available at the PHC clinics. These included:

- National Department of Health Minimum Package of Interventions to Support Linkage to Care, Adherence, and Retention in Care, 2020 version (Minimum Package) [68].
- National User Guide on the Prevention and Treatment of Hypertension in Adults at the PHC Level, 2021 version (User Guide) [21].
- Standard Treatment Guidelines and Essential Medicine List for Primary Healthcare Level, 2020 edition (STG and EML) [69].
- Adult Primary Care (APC) Clinical Tool (2023) [70].
- National Department of Health, 2023: Differential Models of Care in South Africa (Models of Care) [71].

**Figure 2:**
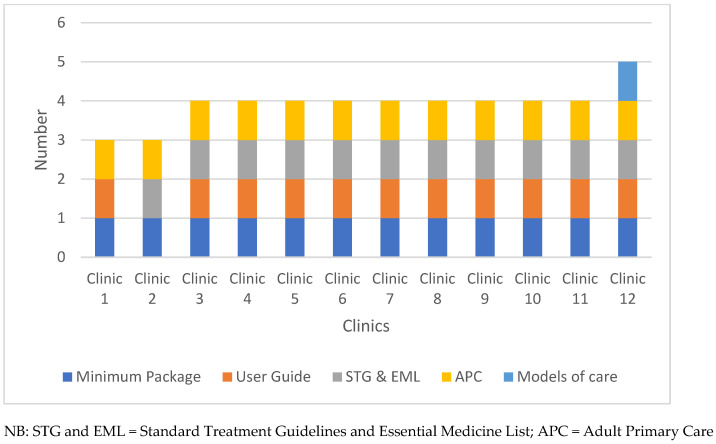
Availability of hypertension guidelines across participating PHC Clinics.

The availability of hypertension management guidelines across the participating clinics is shown in Figure 2.

One clinic reported not using the STG/ EML guideline, indicating it is no longer available in a hard copy, while another clinic reported not having, or using, the User Guide for managing patients with hypertension. The Model of Care guideline was available at only one clinic, where the interviewee served as the operational manager. The minimum package and APC were available at all the clinics (Figure 2).

### 3.4. Access to Hypertension Guidelines at PHC Clinics

**Figure 3:**
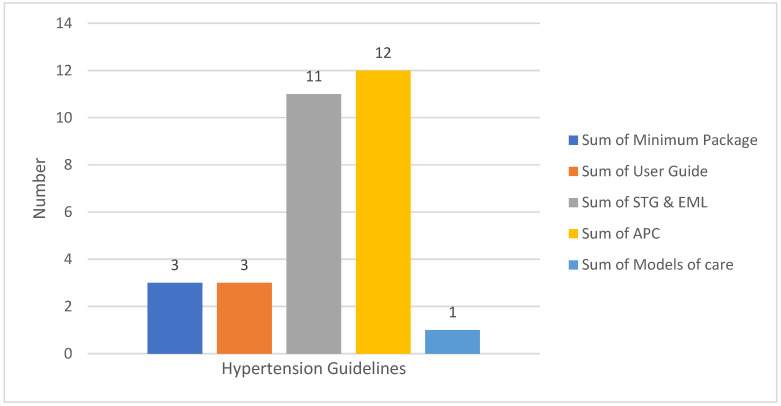
Accessibility of hypertension guidelines during clinical consultations.

The current accessibility of guidelines among participating PHC clinics is shown in Figure 3. (Please refer to Section 2.5 for the definition of accessibility).

The minimum package and the user guidelines were accessible only in three clinics where the participants were facility managers. This is because they were available only on desktops in the manager’s office and could be accessed only in the manager’s presence. The most accessible guidelines were the APC (12 clinics) and STG/EML (11 clinics), both available in hard copy.

### 3.5. Availability of the General Patient Register and the Hypertension Patient Register at the PHC Clinics

All clinics maintained a general patient register, but only two maintained a hypertension-specific register.

### 3.6. Assessment of the Quality of Care for Patients with Hypertension

At least 10 clinics reported assessing the quality of care for hypertensive patients within their facilities in the last 12 months, either internally or externally, by auditing the files of patients with hypertension on a weekly, monthly, or quarterly basis.

### 3.7. Perceptions of the Participants About the Implementation of Quality Indicators

Open-ended questions were coded and grouped into five themes (Table 4).

Some of the themes related to or complemented the qualitative data outcomes. For example, the perceived benefits of quality improvement activities, the effectiveness and self-efficacy of the hypertension quality indicators, and participants had concerns recommendations (Table 4).

## 4. Discussion

We believe this study provides insight into the acceptability of newly agreed quality indicators for managing patients with hypertension among PHC clinics in South Africa and their subsequent use in monitoring adherence to guidelines. In addition, our comprehensive approach also provides preliminary data on the availability and accessibility of hypertension management guidelines currently within PHC clinics in South Africa. Alongside this, offering recommendations for service delivery changes during the implementation of agreed indicators in PHC clinics in South Africa to improve future care. This is important to ensure that any intervention (hypertension quality indicators) enhances the subsequent quality of care and outcomes for patients with hypertension in South Africa given current challenges [17,22,23,32]. Acceptance of potential quality indicators to improve the management of patients with hypertension, especially among nurses at the PHC level, is critical in South Africa and, more broadly, among LMICs, where primary care relies on nurses [22,56,59,66,72].

### 4.1. Exploring the Acceptability of the Quality Indicators and Their Use in Monitoring Adherence to Guidelines

Implementation of hypertension quality indicators would increase the probability of improving the quality of care for these patients. These indicators may also reduce mortality due to hypertension among patients with HIV and AIDS, considering their acceptance among PHC clinics (Table 3 and Table 4). Their acceptance, and their subsequent implementation are important first steps towards successful quality improvement implementation [54,56].

This is consistent with previous studies in which most, or all, participants accepted quality improvement programmes based on process quality indicators [56]. This acceptance should be followed by the implementation of these indicators during regular facility audits. Such audits should drive quality improvement among PHC clinics. This is because when PHC facilities are aware of ongoing audits, they typically strive to prepare for them.

### 4.2. Availability and Access to Hypertension Guidelines

As mentioned, there have been concerns about compliance with current hypertensive guidelines among PHC clinics in South Africa, and other African countries [17,34,35], exacerbated by concerns about their availability and accessibility. Our study found that all participating PHC clinics had hypertension clinical guidelines, and all clinicians were aware of, and had access to, at least one current guideline. This is similar to a previous study in Namibia, in which most prescribers were aware of and had access to the Namibian Standard Treatment Guidelines [73]. However, some guidelines could not be accessed during consultations because they were not available electronically in the consulting room, thereby limiting their use in practice. This also aligns with previous findings of poor compliance with hypertension guidelines in these PHC clinics [23]. This emphasises the need for readily available electronic guidelines for HCPs during consultations to improve their use and, subsequently, patient outcomes [74].

Whilst this study found that all clinics had one or more hypertension guidelines available, their use could not be confirmed, as this was not the study’s objective. However, previous audits, as well as the latest study in these clinics, have found non-compliance with available guidelines [23]. With preliminary data on quality indicators to measure adherence to hypertension guidelines now available, further large-scale studies are needed to assess the extent of and possible reasons for non-adherence to current guidelines to inform future interventions [23,32]. This is particularly important among PHC clinics in South Africa given current concerns with the management of these patients coupled with the government’s goals for patients with CVD.

Of concern is that most PHC clinics in this study lacked a hypertension-specific patient register. This is a challenge given that patients are currently missing review appointments alongside non-adherence to current medications. Going forward, PHC clinics in South Africa need to electronically initiate a patient-specific hypertension register. This would enhance patients’ traceability, improve adherence to patient reviews, and improve prescriber monitoring of agreed indicators.

### 4.3. Recommendations for Implementing Hypertension Quality Indicators at PHC Clinics

Based on our findings, we recommend several follow-up steps, which include specific registers and recall systems (Section 4.3.1, Section 4.3.2, Section 4.3.3, Section 4.3.4 and Section 4.3.5).

#### 4.3.1. Disease-Specific Hypertension Registers in Facilities

This study found that only two of the participating PHC clinics had a hypertension-specific register, which could hinder patients’ traceability and progress monitoring. It is recommended that PHC clinics now develop and implement patient-specific registers for hypertension to facilitate monitoring and tracking of service delivery to patients with this condition. This is important, given the participants’ perceived effectiveness when used with quality indicators (Table 4). Evidence also shows that the hypertension patient registers provide a rich source of patient data, which is typically not available from routine health information systems. This data can also be used to generate funds for its sustainability [75].

An electronically available, patient-specific register, for hypertension would also facilitate tracking of services provided to patients, improve tracing of defaulters, and promote learning from best practices in South Africa. As a result, ensuring that high-quality care is actually provided [39,76].

#### 4.3.2. Develop Recall Systems to Invite Patients to Review Their Progress

Building upon the hypertension patient register, PHC clinics should now develop and implement patient recall systems. This is important for strengthening patient recall and improving attendance at reassessment. Such activities, together with the application of quality indicators, should help improve adherence to current guidelines, a major challenge among PHC clinics including in South Africa [23]. However, this will require implementing electronic data management systems in public PHCs in South Africa (Section 4.3.4).

#### 4.3.3. Training of Primary Care Staff on Quality Assessment/Audit and Quality Improvement

Whilst most clinics reported awareness of quality assessments conducted in the last 12 months, one participating clinic was unaware of any such activities. This is concerning given the Department of Health’s quality initiatives, which included the annual Ideal Clinic projects. This may signal the exclusion of some nurses from quality improvement activities. This is a concern as nurses are typically responsible for delivering quality patient care in PHC facilities in South Africa. Consequently, they should be informed about any quality improvement initiatives as well as their role with improving patient outcomes. Future activities should start with awareness and training of nurses on hypertension guidelines where this is a concern. Subsequently, their involvement with data collection processes and audits, which includes monitoring patient outcomes for reporting purposes. In addition, helping with any legal requirements from the various statutory bodies responsible for providing quality healthcare to the public in South Africa [33,36,45,74,77].

Future research will though be needed to explore collaborations between the Department of Health and the various health regulatory bodies in South Africa. The objective is to make patient quality-of-care improvement interventions a continuous professional development initiative, instigated and monitored by institutions of higher learning. This is a requirement to enhance HCP awareness and subsequent competency with improving the quality of care for patients with hypertension in PHC clinics throughout South Africa.

#### 4.3.4. Transform Data Management to Electronic E-Health Systems

Participants in this study highlighted concerns regarding manual data management, and its subsequent burden on nurses, to continually monitor patient care. A previous study found poor documentation and a lack of data at PHC clinics in South Africa [22]. This may be related to manual documentation practices, exacerbated by insufficient numbers of HCPs at these clinics. As a result, there needs to be a rapid transition from typically manual documentation practices to electronic health systems for data management among all PHC clinics throughout South Africa. Currently, there is only partial electronic data capture for some elements in the District Health System. This is important because the transformation of information systems is central to delivering quality healthcare at the PHC level [78]. This would enhance data quality to inform future clinical and policy decisions, thereby improving outcomes among patients with hypertension [78]. Such needs highlight the importance of an equal focus on healthcare facilities and information systems during every patient visit in South Africa and beyond. However, this must be predicated on a regular and reliable supply of electricity alongside a continuous internet connection, which is currently a concern in South Africa.

#### 4.3.5. Adherence to an Agreed Hypertension Guideline

Encouragingly, more than one hypertension guideline was found in each participating clinic. However, previous audits in these clinics have found non-compliance with the guidelines (Table 4). This is a concern, given the South African government’s aim of achieving an 80% control of blood pressure among patients on treatment at the PHC level by 2030 [11]. In addition, the current situation indicates that this goal remains unattainable due to poor-quality care in PHC clinics [17,23]. Consequently, it becomes critical to improve the quality of care of patients with hypertension by encouraging HCPs to adhere to current hypertension guidelines when managing these patients. It is recommended going forward that PHC clinics in South Africa now adopt a single, specific set of hypertension guidelines and key quality indicators for use in their clinics to facilitate data comparison, improve monitoring, and promote the use of the latest evidence to improve the quality of care [16]. This ongoing research will assist with this.

**Figure 4:**
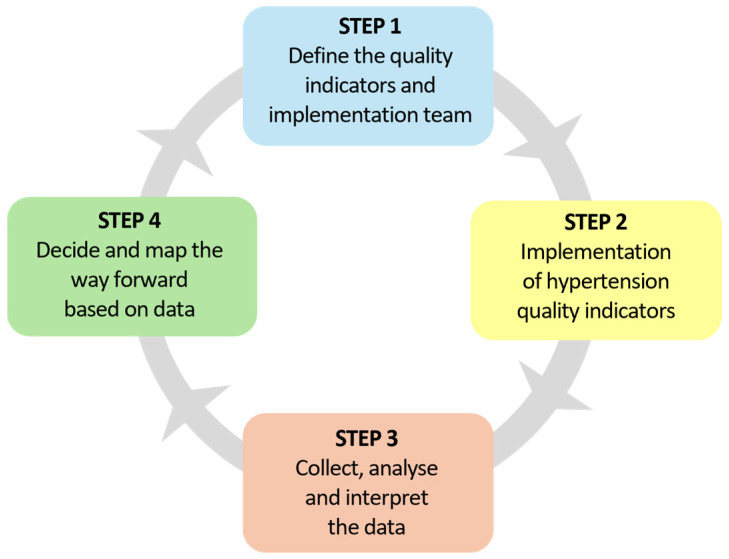
Process of implementation of the recommendations [79].

In view of our findings, we recommend a step-by-step approach following the Plan-Do-Study-Act (PDSA) cycle to implement these recommendations. The inclusivity of all stakeholders in quality improvement is crucial for its successful implementation. Figure 4 illustrates the repeatable cycle for implementing the improvement plan.

### 4.4. Strengths and Limitations

A strength of this study was that only one experienced researcher undertook the interviews and collected the data, providing consistency. In addition, the questionnaire was based on the previously validated TFA model to assess acceptability and was piloted to test its feasibility in the study population, thereby reducing ambiguity.

We recognise that the study has several limitations. These include only going to 12 PHCs for the reasons stated. However, this study was exploratory implementation research [65,80] generating preliminary acceptability and contextual feasibility insights. Consequently, it cannot be used to generalise the findings. Rather it was a translational study aimed at providing insight into the context and barriers to implementing hypertension quality indicators from the perspective of HCPs working in public PHCs in South Africa.

Alongside this, whilst managers and facility managers identified prospective participants based on their roles, in some cases, only one was identified. This may introduce selection and social desirability bias. However, this was accounted for by aggregating the average scores across the four Likert-rated questions and the three acceptability constructs. We believe that any bias was also minimised by providing participants with equal opportunities after their introduction to the researcher. If one participant in any designated PHC could not take part for any reason, the next participant was invited to participate, with arrangements to return on another day when a new group of nurses was available.

Interviewer-led open-ended questioning may also have introduced inconsistency and potential influence on participants. However, this was minimised by using the same researcher throughout the study, a semi-structured questionnaire for data collection, and allowing participants to record their responses.

This study aimed to explore the real-world application of the indicators and not any direct association with clinical outcomes. While the presence of higher quality structures and acceptability, as well as application of guidelines does not automatically translate into effectiveness or improved patient outcomes, it provides increased probability of doing so [81].

Despite these limitations, we believe this study provides valuable insights into HCPs’ acceptance of, and concerns about implementing hypertension quality indicators at the PHC level in South Africa. Alongside this, recommendations and inputs from those who implement quality improvement programmes informed the changes that will be needed to fully apply the indicators among all PHC facilities in South Africa.

## 5. Conclusions

This study aimed to explore the acceptability of hypertension quality indicators and their use for monitoring guideline adherence. In addition, it aimed to assess the routine availability and accessibility of hypertension management guidelines among PHC clinics. Alongside this, we aimed to identify recommendations for service delivery changes that may be needed in PHC clinics across South Africa to improve the quality of hypertension care.

Quality indicators can be used to monitor adherence to hypertension guidelines. Subsequently, they are used to improve the quality of care and patient outcomes at the PHC level in South Africa, while also addressing other pertinent issues.

Implementing hypertension quality indicators across all PHC clinics in South Africa, alongside investments in electronic information management and further human resource assessments are the next necessary steps to improve hypertension management at the PHC level. Future research is necessary to assess the feasibility of implementing hypertension quality indicators in other LMICs to harmonise data and facilitate comparison within the region. We will continue to monitor the situation.

## Figures and Tables

**Table 1:** Hypertension quality indicators for South African PHC facilities.

Percentage of patients in the practice/unit/facility with a BP recorded in the last 12 months Patient with BMI recorded in the past 12 months Patient had serum potassium concentration recorded in the past 6 months for patients on spironolactone or eGFR < 30 mL/min Patient had serum creatinine concentration and eGFR recorded in the past 12 months for patients with proteinuria of 1+ or more Patient had serum creatinine concentration and eGFR recorded in the past 12 months for patients with existing cardiovascular disease Patient had serum creatinine concentration and eGFR recorded in the past 12 months for patients with hypertension for 10 years or more Patient had serum creatinine concentration and eGFR recorded in the past 12 months for patients with uncontrolled hypertension Patient had serum creatinine concentration and eGFR recorded in the past 12 months for patients with chronic kidney disease (eGFR < 60 mL/min) Patient had finger-prick blood glucose recorded in their medical record in the past 12 months Patient had urine protein by dipstick in the past 12 months Patients in the practice/unit/facility were screened for cardiovascular disease risk factors in the last 12 months Patient in the practice/unit/facility checked for medicines and lifestyle modification adherence before escalating therapy Patient aged 40 years and over with a BP measurement recorded in the preceding 5 years Patient had a hypertension review with a doctor recorded in the last 12 months Patient who had a hypertension review with a nurse/doctor recorded in the past 6 months after the BP is controlled, for patients with uncontrolled BP Patient had a hypertension review with a nurse/doctor recorded in their medical record one month after being in step 7 of the algorithm of hypertension management, for patients already on medication Patient with a new diagnosis of hypertension aged 18–84 years, recorded (excluding those with pre-existing CHD, stroke and/or TIA), who had a recorded CVD risk assessment score of >20% in preceding 12 months Patients who are currently treated with statins (unless there is a contraindication) in those patients with a new diagnosis of hypertension aged 18–84 years, recorded (excluding those with pre-existing CHD, stroke and/or TIA), who had a recorded CVD risk assessment score >20% in the preceding 12 months Patient with hypertension aged 18 to 74 years in whom there was an annual assessment of physical activity in the preceding 15 months Patient in the practice/unit/facility who has been counselled about the importance of smoking cessation in the last 12 months Patient in the practice/unit/facility who has been counselled about the importance of maintaining ideal body weight, i.e., BMI < 25 kg/m^2^, in the last 12 months Patient in the practice/unit/facility who has been counselled about the importance of salt restriction with increased potassium intake from fresh fruits and vegetables in the last 12 months Patient in the practice/unit/facility who has been counselled about the importance of reducing alcohol intake to no more than 2 standard drinks per day for males and 1 for females in last 12 months Patient in the practice/unit/facility who has been counselled about to follow a healthy eating plan in the last 12 months Patient records with evidence that the nurse/doctor counselled the patient on the importance of engaging in physical activity, eating small portions of healthy food, using less salt, using alcohol in moderation, stopping smoking, reducing stress, committing to take medication regularly Patient in the practice/unit/facility who has been counselled about the importance of engaging in regular moderate aerobic exercise, e.g., 40 min brisk walking at least 3 times a week, in the last 12 months Patients diagnosed with hypertension who were given lifestyle advice in the preceding 12 months for smoking cessation, safe alcohol consumption, and healthy diet Patient with hypertension and a BMI of ≥27.5 kg/m^2^ or ≥30 kg/m^2^ in the preceding 12 months referred to a weight management programme within 90 days of the BMI being recorded Patient had cholesterol recorded in the last 12 months Patients had heart/pulse recorded in the last 12 months Patient had heart/pulse recorded in their medical record in the last 6 months Patient had random blood glucose (≥11.1 mmol/L)/fasting blood glucose (≥7.0 mmol/L) recorded in past 6 months for all adult patients who are >40 years old and who are overweight (BMI > 25) or obese (BMI > 30) Patient tested for the presence of protein in the urine by sending a urine sample for estimation of the albumin–creatinine ratio in the last 12 months Patients with a new diagnosis of hypertension who have a record of a test for haematuria in the three months before or after the date of entry to the hypertension register Patients with a new diagnosis of hypertension who have a record of urinary albumin–creatinine ratio test in the three months before or after the date of entry to the hypertension register Patient with a BP of <140/90 mmHg with no adverse medicine reactions in patients who are in step 2 of the algorithm of hypertension management for patients already on medication every six months Patient with a BP of <140/90 mmHg with no adverse medicine reactions in patients who are in step 3 of the algorithm of hypertension management for patients already on medication Patient with a BP of <140/90 mmHg with no adverse medicine reactions in patients who are in step 4 of the algorithm of hypertension management for patients already on medication every six months Patient with a BP of <140/90 mmHg with no adverse medicine reactions in patients who are in step 5 of the algorithm of hypertension management for patients already on medication every six months Patient with a BP of <140/90 mmHg with no adverse medicine reactions in patients who are in step 6 of the algorithm of hypertension management for patients already on medication every six months Patient with a BP of <140/90 mmHg with no adverse medicine reactions in patients who are in step 7 of the algorithm of hypertension management for patients already on medication every six months Patient referred to the doctor/district level services in the last 12 months Patient had referral and reasons for referral in the last 12 months Pregnant patients who were referred to district hospital services because they had severe pre-eclampsia and imminent eclampsia Patient received all core CVD/hypertension drugs

NB: BMI, body mass index; BP, blood pressure; CHDs, coronary heart diseases; CVD, cardiovascular disease; eGFR, estimated glomerular filtration rate; TIA, transient ischemic attack. Adapted from [23].

**Table 2:** Characteristics of PHC clinics (*n* = 12).

Clinic and Patient Characteristics	Units
Rural facility; *n*	10
Facility patient register in use; *n*	12
Conducted quality assessment in the last 12 months; *n*	10
Average age of hypertension patients seeking care at the facility; years	44
Average number of hypertension patients seen per week; *n*	121

**Table 3:** Participants’ perceptions of the acceptability of hypertension quality indicators (*n* = 12).

Statement	Agree (*n*)	Neutral (*n*)	Disagree (*n*)
Implementation of hypertension indicators would be fair to the facility	12	0	0
The implementation of hypertension indicators will improve the management of hypertension patients in your facility	12	0	0
Implementation of the hypertension indicators will interfere with your other priorities	1	0	0
Implementation of the hypertension indicators will be time-consuming for you	3	1	8

**Table 4:** Descriptive categorisation of themes from content analysis.

Theme	Frequency	Example Quote
Participants’ experience with quality assessments and improvement activities	3	“Audit the clinic and files for Ideal clinic assessment, and we were advised to do BMI and heart rate on every patient.” (Participant 06)
Benefits of quality improvements activities	6	“Internal audit to classify defaulters and any test conducted. Patients with controlled blood pressure, we enrolled them on Central chronic medicines dispensing and distribution.” (Participant 02)
Perceived effectiveness and self-efficacy	7	“The hypertension patient register would make a huge difference in determining if patients are receiving the treatment for hypertension. Indicators would add to what is in place at the moment.” (Participant 03)
Concerns	2	“We are not well-staffed, which can interfere with other priorities.” (Participant 05)
Recommendations	2	“Make it user-friendly electronically.” (Participant 04)

## Data Availability

Further inquiries can be directed to the corresponding authors.

## Supplementary Materials

The following supporting information can be downloaded at https://www.mdpi.com/article/10.3390/healthcare14121666/s1, Supplementary Table S1 contains the final list of 22 quality indicators that could currently be applied to ≥75% of the population for monitoring hypertension management at the PHC level in South Africa [23]. Supplementary Table S2 contains details of the consent form.
