# Supplementary material for: Acceptability of Hypertension Quality Indicators in Primary Care in South Africa: Exploratory Implementation Research
Source: Healthcare (Basel). 2026 Jun 11;14(12):1666. doi: 10.3390/healthcare14121666 (PMC13299701; doi:10.3390/healthcare14121666)
Supplement: Supplementary file 1 [file healthcare-14-01666-s001.zip › healthcare-4283656-supplementary.pdf]

Supplementary Tables

Table S1 – 22 Hypertension quality indicators applied to ≥ 75% of the population among PHCs in South Africa

No.	Indicator
1.	Percentage of patients in the practice/unit/facility with a BP recorded in the last 12 months
2.	Patient with BMI recorded in the past 12 months
3.	Patients had finger-prick blood glucose recorded in their medical record in the past 12 months
4.	Patient has had urine protein by dipstick in the past 12 months
5.	Patients in the practice/unit/facility were screened for cardiovascular disease risk factors in the last 12 months
6.	Patient aged 40 years and over with a BP measurement recorded in the preceding 5 years
7.	Patient had a hypertension review with a doctor recorded in the last 12 months
8.	Patient who has a hypertension review with a nurse/doctor recorded in the past 6 months after the BP is controlled, for patients with uncontrolled BP
9.	Patient with hypertension aged 18 to 74 years in whom there was an annual assessment of physical activity in the preceding 15 months
10.	Patient in the practice/unit/facility who had been counselled about the importance of smoking cessation in the last 12 months
11.	Patient in the practice/unit/facility who has been counselled about the importance of maintaining ideal body weight, i.e., BMI < 25 kg/m^2, in the last 12 months
12.	Patient in the practice/unit/facility who has been counselled about the importance of salt restriction with increased potassium intake from fresh fruits and vegetables in the last 12 months
13.	Patient in the practice/unit/facility who has been counselled about the importance of reducing alcohol intake to no more than 2 standard drinks per day for males and 1 for females in last 12 months
14.	Patient in the practice/unit/facility who has been counselled about to follow a healthy eating plan in the last 12 months
15.	Patient records with evidence that the nurse/doctor counselled the patient on the importance of engaging in physical activity, eating small portions of healthy food, using less salt, using alcohol in moderation, stopping smoking, reducing stress, committing to take medication regularly
16.	Patient in the practice/unit/facility who has been counselled about the importance of engaging in regular moderate aerobic exercise, e.g., 40 min brisk walking at least 3 times a week, in the last 12 months
17.	Patient had cholesterol recorded in the last 12 months
18.	Patients have heart/pulse recorded in the last 12 months
19.	Patient had heart/pulse recorded in their medical record in the last 6 months

No.	Indicator
20.	Patient tested for the presence of protein in the urine by sending a urine sample for estimation of the albumin–creatinine ratio in the last 12 months
21.	Patient referred to the doctor/district level services in the last 12 months
22.	Patient received all core CVD/hypertension drugs

NB: Adapted from Rampamba et al (2025) [23]

Supplementary Table S2 - Consent form

SEFAKO MAKGATHO HEALTH SCIENCES UNIVERSITY ENGLISH CONSENT FORM

You are hereby kindly invited to participate in a Research Project

Name of Project: **Development of a Quality Indicator Framework for Hypertension at Primary Health Care Level in South Africa**

I have read the information on/heard the aims and objectives of the proposed research project and was provided the opportunity to ask questions and given adequate time to rethink the issue. The aim and objectives of the project are sufficiently clear to me. I have not been pressurized to participate in any way.

I know that I will have to complete the questionnaire. I am aware that this material may be used in scientific publications which will be electronically available throughout the world. I consent to this provided that my name is not revealed.

I understand that participation in this Study is completely voluntary and that I may withdraw from it at any time and without supplying reasons.

I know that this Study has been approved by the Sefako Makgatho University Research Ethics Committee (SMUREC), Sefako Makgatho Health Sciences University. I am fully aware that the results of this Study will be used for scientific purposes and may be published. I agree to this, provided my privacy is guaranteed.

I hereby give consent to participate in this Study.

....

Name of participant Signature of participant

....

Place Date Witness

Statement by the Researcher

I provided verbal and/or written* information regarding this Study
I agree to answer any future questions concerning the Study as best as I am able.
I will adhere to the approved protocol.

Enos Muisaphanda Rampamba ____________ ____________ ____________

Name of Researcher Signature Date Place
